# The CD44^high^ Tumorigenic Subsets in Lung Cancer Biospecimens Are Enriched for Low miR-34a Expression

**DOI:** 10.1371/journal.pone.0073195

**Published:** 2013-09-03

**Authors:** Saroj K. Basak, Mysore S. Veena, Scott Oh, Chi Lai, Sitaram Vangala, David Elashoff, Michael C. Fishbein, Sanjai Sharma, Nagesh P. Rao, Dinesh Rao, Ryan Phan, Eri S. Srivatsan, Raj K. Batra

**Affiliations:** 1 Wadsworth Stem Cell Institute, Veterans Affairs Greater Los Angeles Healthcare System (VAGLAHS), Los Angeles, California, United States of America; 2 Pathology and Laboratory Medicine, David Geffen School of Medicine at UCLA, Los Angeles, California, United States of America; 3 Jonsson Comprehensive Cancer Center, David Geffen School of Medicine at UCLA, Los Angeles, California, United States of America; 4 Division of General Surgery/Department of Surgery, David Geffen School of Medicine at UCLA, Los Angeles, California, United States of America; 5 Department of Medicine, David Geffen School of Medicine at UCLA, Los Angeles, California, United States of America; University of Pittsburgh, United States of America

## Abstract

Cellular heterogeneity is an integral part of cancer development and progression. Progression can be associated with emergence of cells that exhibit high phenotypic plasticity (including “de-differentiation” to primitive developmental states), and aggressive behavioral properties (including high tumorigenic potentials). We observed that many biomarkers that are used to identify Cancer Stem Cells (CSC) can label cell subsets in an advanced clinical stage of lung cancer (malignant pleural effusions, or MPE). Thus, CSC-biomarkers may be useful for live sorting functionally distinct cell subsets from individual tumors, which may enable investigators to hone in on the molecular basis for functional heterogeneity. We demonstrate that the CD44^hi^ (CD44-high) cancer cell subsets display higher clonal, colony forming potential than CD44^lo^ cells (n = 3) and are also tumorigenic (n = 2/2) when transplanted in mouse xenograft model. The CD44^hi^ subsets express different levels of embryonal (de-differentiation) markers or chromatin regulators. In archived lung cancer tissues, ALDH markers co-localize more with CD44 in squamous cell carcinoma (n = 5/7) than Adeno Carcinoma (n = 1/12). MPE cancer cells and a lung cancer cell line (NCI-H-2122) exhibit chromosomal abnormalities and 1p36 deletion (n = 3/3). Since miR-34a maps to the 1p36 deletion site, low miR-34a expression levels were detected in these cells. The colony forming efficiency of CD44^hi^ cells, characteristic property of CSC, can be inhibited by mir-34a replacement in these samples. In addition the highly tumorigenic CD44^hi^ cells are enriched for cells in the G2 phase of cell cycle.

## Introduction

Tumor heterogeneity can be characterized by differential expression of cell surface markers, genetic and epigenetic differences, and/or differences in key signaling molecules or effectors of cell function. Cellular heterogeneity can be characterized by differences in the functional (behavioral) properties of cells (clonogenicity, colony formation ability in soft agar, tumorigenesis etc.). Whereas many investigations have opted to associate cell surface markers in tumor cells found at the primary tumor site with CSC-behavioral properties, we observed that clinically advanced stages are particularly enriched for cell subsets bearing CSC-biomarkers. Thus, we postulated that advanced stage disease does not prohibit (and may be advantageous) for associating specific biomarkers with functional phenotypes. Accordingly, our approach to biological discovery emphasizes designing appropriate functional bioassays to characterize both the cell phenotypes and molecular biology underlying tumor initiation, as well as tumor progression.

Lung cancer is the leading cause of cancer mortality in both men and women; with non small cell lung cancer (NSCLC) accounting for 80–85% of cases [1]. For comprehending the biology underlying this high mortality, we have selected an advanced stage disease model (MPE). Lung cancer patients presenting with MPE have significantly higher mortality than those without MPE, or those who have cytologically negative effusions [2]–[4]. Thus, the MPE-tumor burden is imbued with biological properties that diminish survival of cancer patients. Importantly, the MPE bulk tumor population is comprised of heterogeneous subpopulations [5]. In part, this heterogeneity can be characterized by biomarkers typically associated with features of CSC (CD44, ALDH, cMET, CD166, MDR-1, uPAR, PTEN, OCT-4, BMI-1, hTERT, SUZ12, EZH2).

An objective of the present study was to determine if we could identify a tumor cell subset that displayed an increased competence for tumor propagation and maintenance, and to begin to characterize the molecular bases for these properties. We first studied CD44 as a selection marker for cells predicted to have high tumorigenic potential because it has previously identified CSC in various epithelial cancers, including breast [6], head and neck, [7], [8], pancreatic [9], [10], and prostate malignancies [11]–[15]. CD44 is highly expressed in different lung cancer subtypes, [16], and its expression is related to poor prognosis in patients [17]. Recent studies in NSCLC cell lines also characterize CD44^hi^ cells as CSC [16].

MPE-primary cultures contain a subpopulation of cells that highly expresses CD44 (CD44^hi^). When these cells are sorted from the MPE-primary cultures, they exhibit high tumorigenic potential, including engraftment of tumors in NOD/SCID IL2γR^null^ mice in limiting dilutions of cell transplants. These properties are characteristic of CSC. Fractions of CD44^hi^ cells are associated with an elevated expression of another CSC-marker associated with xenobiotic metabolism, ALDH. The CD44^hi^/ALDH^hi^ phenotype is evident in both squamous cell (SCC) and adenocarcinoma (AC) of the lung, suggesting that similar marker profiles may label behaviorally aggressive (highly tumorigenic) cell fractions across the various “lineages” (histopathological subtypes) of lung cancers [18].

MPE tumors commonly display hyperploidy and chromosomal abnormalities. FISH analysis detected a common specific abnormality in 1p36 region, suggesting that this region may play an important role in contributing to aggressive behavioral properties. The 1p36 region has previously been identified for containing the locus that encodes tumor suppressor microRNA (miR-34a). Loss of miR-34a expression is implicated in cancer progression [15], [19]; this study adds to that evidence. Highly tumorigenic CD44^hi^ cells express low miR-34a, and miR-34a replacement inhibits colony formation of CD44^hi^ cells in soft agar. The cell cycle analysis of CD44^hi^ cells indicated that these highly tumorigenic cells reside in the G2 phase of cell cycle.

## Materials and Methods

### Malignant Pleural Effusion (MPE) Collection, Processing and Cell Culture

All subjects in the study underwent written informed consent by a process approved by the institutional review board (IRB) at the Veterans Affairs-Greater Los Angeles Healthcare System (VAGLAHS) and the study was approved by IRB-VAGLAHS. MPE specimens (M-1, M-2 and M-3) were collected from patients at Veterans Affairs-Greater Los Angeles Healthcare System (VAGLAHS). Cells are cultured in presence of 20–30% MPE (primary culture medium or PCM) as described previously [5]. (Supporting Information S1 and S2).

### Control Established Cell Lines

Two established cell lines GM 05399 (normal fibroblast) and H2122 (lung cancer) were used in the study. The fibroblast cell line GM 05399 was obtained from the Coriell Institute for Medical Research (Camden, NJ). The cell line was derived from a 1-year old Caucasian male. The cell line is maintained in our laboratory in Dulbecco’s Modified Eagle’s Medium (DMEM) in presence of 10% fetal bovine serum (FBS) [20]. The H2122 lung adenocarcinoma cell line was generated by Adi Gazdar from a malignant pleural effusion, and acquired from Ilona Linnoila and Herb Oie from the NCI. It was subsequently deposited into ATCC (NCI-H2122 [H2122] ATCC® CRL-5985™) [21]. The cell line is maintained in our laboratory in RPMI-1640 medium in presence of 10% FBS [22], [23]. Both the cell lines are publicly available.

### Antibodies

The following antibodies were used for flow cytometry FACS/Sort: Mouse anti-Human IgG2b CD44-FITC, (BD Biosciences # 555478); FITC Mouse IgG2b κ Isotype control, BD Biosciences # 555742); PE-labeled mouse anti-human CD44, (BD Pharmingen # 555479); PE Mouse Mouse IgG2b κ Isotype control, BD Biosciences 555743. Anti-CD166-FITC (Mouse monoclonal; IgG1 Setrotech # MCA 1926F, primary unlabeled anti-cMET (mouse IgG2a, Abcam # 49210), anti-uPAR (mouse IgG, Santa Cruz Biotech # 13522), Secondary antibody used for the study used were: Goat (Fab’)2 anti-Mouse IgG (H+L)-PE-Cy.5.5 (Caltag laboratories # M35018).

### Immunohistochemistry (IHC)

Primary human lung cancer tissue (squamous cell carcinoma: SCC and adenocarcinoma: AC) or human lung control tissue (human normal alveolar and bronchiolar tissues) were obtained from the UCLA Department of Pathology core facility. Xenograft tumors derived from CD44^hi^ cells injected in NOD/SCID (IL2rγ^null^) mice were surgically removed, cut into 0.3–0.5 mm pieces and fixed in ethanol (Fisher Scientific) or Z-fix (Anatech, MI). For IHC, sections 3–5 µ sections were cut and deparaffinized and processed for antigen retrieval [5] and stained for marker expression. Initially tissue sections were stained with single marker antibody staining (CD44 or ALDH). Once the conditions were optimized for single antigen staining then the dual antigen staining (CD44 and ALDH) of tissue sections was achieved. Paraffin-embedded tissue sections were deparaffinized and rehydrated. After antigen retrieval (10 mM sodium citrate buffer, PH 6.0 by steam 25 minutes) and blocking, endogenous peroxidases were quenched (3% H_2_O_2_ in 1% sodium azide with PBS, 30 minutes in room temperature). Slides were incubated with primary rabbit polyclonal antibody to ALDH1A1 (Abcom Inc. Cat# ab51028), overnight at 4°C. The slides were washed with PBS and incubated with EnVision+ System-HRP Labelled Polymer Anti-Rabbit (Dako Cat# K4003) for 30 minutes. The slides were incubated in DAB (Vector Peroxidase Substrate Kit #SK-4100 with Nickel Sol) for 10–20 minutes and then the slides were washed 5 minutes 3 times with PBS. For double staining with CD44 (R & D Systems, mouse monoclonal IgG, Cat# BBA 10), slides were also incubated in the primary antiserum at room temperature for 1 hour, followed by the secondary antibody, Biotinylated-anti-mouse IgG (Vector Cat# 9200), and then, ABC kit (Vector Cat# AK-5000) and Vector Red Alkaline Phosphatase Substrate Kit I (Vector Cat# SK-5100), developed for 20 minutes. Sections were counter-stained with Harris’ hematoxylin, dehydrated in graded alcohol, cleared in xylene and mounted on glass slides with cover slip. The stained sections were examined under a microscope (Leica-Leitz DMRBE or Olympus 1×71) and positive or dual antigen expressing areas determined by pathologists at UCLA.

### Cytology and Flow Cytometry (FACS)

Photomicrographs were taken using the Leica- Leitz DMRBE microscope mounted with a CCD camera and FACS analysis was done using the Becton Dickinson FACSCalibur Analytic Flow Cytometer [5]. Cell sorting was performed using the Becton Dickinson FACSVantage SE Sorting Flow Cytometer at the UCLA-JCCC Flow-cytometry core facility.

### Reverse Transcriptase- PCR (RT-PCR) Analysis of Gene Expression

The primary samples were first sorted into CD44^hi^ and CD44^lo^ populations. The cells were collected and RNA was extracted using Trizol and Fast Track 2.0 mRNA isolation kit (Invitrogen Inc., Carlsbad, CA) and was reverse transcribed using RT kit [5]. The samples were used for PCR for the amplification of Bmi1, hTERT, SUZ12, EZH2, and Oct4 genes. The following primers were used: *Bmi1* Forward - 5′ AATCTAAGGAGGAGGTGA 3′, Reverse- 5′ CAAACAAGAAGAGGTGGA 3′, *hTERT* Forward -5′ GGAATTCTGGAGCTGCTTGGGAACCA 3′, and Reverse- 5′ CGTCTAGAGCCGGACACTCAGCCT-TCA 3′, *SUZ12* Forward -5′ GATAAAAACAGGCGCTTA-CAGCTT 3′, and Reverse-5′ AGGTCCCT-GAGAAAATGTTTCGA 3′, *EZH2* Forward- 5′ TTGTTGGCGGAAGCGTGTAAAATC 3′, and Reverse -5′ TCCCTAGTCCCGCGC-AATGAGC -3′, and Oct4 Forward- 5′ CAACTCCGATGGGGCCCT 3′, and Reverse -5′ CTTCAGGAGCTTGGCAAATTG 3′. The conditions for amplifications of different genes have been described previously [5]. PCR products were separated by 8% gels (TBE, 50 mM Tris borate pH 8.0, 1 mM EDTA) followed by Ethidium Bromide staining. Gels were analyzed using the Kodak 1D software.

### Colony Formation Efficiency Assay

*In vitro* colony-formation assays were done as described [12]. Sorted CD44^hi^ and CD44^lo^ cells were plated at clonal density (100–500 cells/well) in six well tissue culture dishes in triplicates. Holoclones with >20 cells were counted at the end of 10 days of culture. The results are expressed as percentage cloning efficiency.

### Spheroid Formation in Soft Agar Assay

Sorted CD44^hi^ and CD44^lo^ cells were plated at 1000 cells/well in triplicates in six-well culture plates containing 0.35% top agar layered over 0.5% base agar (DNA Grade) containing PCM. Colonies were counted at 3 weeks post plating, results represent mean from three independent experiments.

### Tumorigenicity in NOD/SCID (IL2rγ^null^) Mice

All mice work related protocol for the study was approved by the Institutional Animal Care and Use Committee at UCLA/VAGLAHS. CD44^hi^ and CD44^lo^ cells were sorted by FACS and injected at different cell doses (300/mouse, 3000/mouse and 30000/mouse) at the right and left flank respectively in NOD/SCID (IL2γ^null^) mice in 100 µl of saline. Mice were monitored for tumor growth at both the flanks. Results are represented as group averages of tumor volume, as described [24].

### miR-34a Transfection Studies

To analyze the effects that miR-34a has on colony formation efficiency in soft agar assay the CD44^hi^ cells were transiently transfected with either miR-34a (AM17100, Applied Biosystem/Ambion) or the negative control (scrambled) oligonucleotide. Similarly CD44^lo^ cells were transiently transfected with either anti-miR-34a inhibitor (#AM17000, Applied Biosystem/Ambion) or negative control anti-miR oligonucleotide (#AM17010, Applied Biosystem/Ambion). The transfection was carried out with CD44^hi^ or CD44^lo^ cells using Lipofectamin 2000 (Invitrogen) in 6 well plates with 50,000 cells/well with 100 pmol of miR, anti-miR and control scrambled/oligonucleotides. After 2 days of transfection the cells were collected and assayed for soft agar colony forming efficiency as described above.

### Fluorescent in situ Hybridization (FISH) Analysis of MPE Samples

FISH studies were performed according to established protocol [25]. LSI 1p36 probe was labeled with spectrum orange and LSI 1q25 probe was labeled with spectrum green and hybridized to metaphase spreads as previously described [25], [26]. Briefly, metaphase spreads were prepared by standard cytogenetic procedures. Labeled probes were hybridized and washes were performed under identical conditions of stringency. Slides were hybridized at 37°C overnight with 1–4 ng of the probe, 50% formamide, 10% dextran, 2× SSC, and 50 ng Cot 1 DNA to suppress repetitive sequences. Metaphase chromosomes were counterstained with 4,6-diamidino 2-phenylindole (DAPI) in Vectashield solution (Vector Laboratories Inc., Burlingame, CA). Karyotyping of chromosomes were performed according to established protocols.

### Reverse Transcriptase- Quantitative PCR (RT-qPCR) Detection of mir-34a in MPE Samples

Total RNA was isolated from samples using TRIzol. miR-34a was measured by Step One Plus Real-time PCR system (Applied Bio systems, CA) by using Taq-Man MicroRNA Assays (Applied Biosystems, Foster City, CA) and normalized by RNU48 levels. 3 ul of 20 ng/ul of total RNA was used to perform Reverse Transcriptase (RT) reaction (30 min at 16 deg, 30 min at 42 deg, 5 min at 85 deg) using 10 mM dNTPs, MultiscribeRT enzyme, 10× RT buffer, RNase inhibitor, Taqman RT primer and water in total reaction volume of 15 ul. For qPCR, 10 ul of 2× Taqman universal PCR master mix (No AmpErase UNG from ABI), 7 ul of water, 1 ul of Taqman primer (miR-34a and RNU48) and 2 ul for cDNA for each reaction was used, following amplification protocol (10 min at 95 deg, 15 sec for 95 deg, 60 sec at 60deg for 40 cycles) using Step One Plus Real-time PCR system (Applied Biosystems, CA).

### Surface Marker Labeling and Cell Cycle Analysis

Cells were stained with CD44-FITC and PI (Propidium Iodide) for cell cycle analysis (modified from UCLA/Flow-cytometry core facility protocol). Briefly, 1×10^6^ single cell suspension was washed with PBS/2%PCM, pelleted, and labeled with mouse anti-Human IgG2b CD44-FITC antibody (BD Biosciences # 555478) for 45 min. at room temperature in dark, control antibody was used as negative control. The samples were re-suspended in 1 ml of buffer containing 10 micrograms/ml of PI and 11.25 Kunitz units of RNase and incubate for at least 30 min at 4°C in the dark and analyzed on the flow cytometer within 30 min of PI staining.

### Statistical Analysis

Data are represented as mean±SD and were analyzed with two-sided *t* test by EXCEL and repeated measures analysis of variance (ANOVA) was used for comparison among groups by SAS 9.3. A *P* value<0.05 was considered statistically significant.

## Results

### CD44 Expression Profile of MPE Derived Tumor Cells

MPE-tumor cells can be isolated and expanded in short term primary cultures in presence of MPE fluid and autologous non-tumor cells [5]. Heterogeneous populations, including candidate CSC, are present in the MPE- tumor population, as reflected by the variable expression of CSC-biomarkers: c-MET, uPAR, MDR1, CD166, CD44, and ALDH. Thus, in addition to *intra*tumoral morphological heterogeneity, there are differences in the surface CD44 labeling intensities, and these differences can be exploited to segregate cell subsets [5].

**Figure 1:**
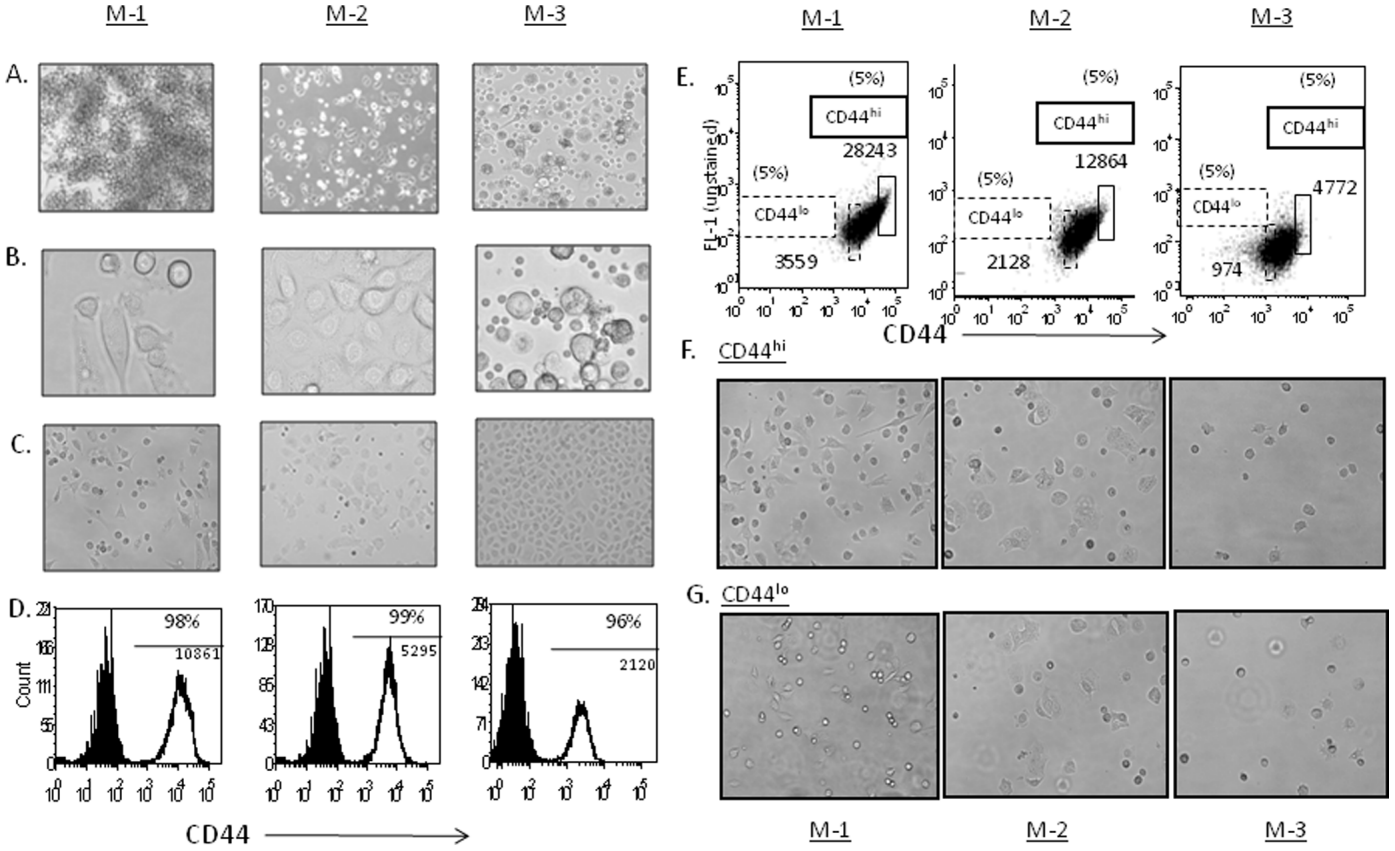
Morphologically variant cells in MPE samples and absence of morphological changes between CD44^hi^ and CD44^lo^ cells. The tumor cells from M-1, M-2 and M-3. (**A**) 100× (2–3 weeks). (**B**) 400× (2–3 weeks). (**C**) Later stages of culture 100× (6–10 weeks). (**D**) CD44- FACS expression pattern and MFI. (**E**) Sorting of CD44^hi^ and CD44^lo^ cells (5–10%). The sorted cells CD44^hi^ and CD44^lo^ were washed and plated out in PCM for 2–3 days to evaluate their morphological differences. (**F**) Morphology of sorted CD44^hi^ cells and sorted (**G**) CD44^lo^ cells were similar (100×). The purity of the CD44^hi^ and CD44^lo^ cells were ≥98%, as revealed by post sort analysis (data not shown).

The primary cultures from three different MPE-samples (M-1, M-2 and M-3), contain morphological variants (flat, oval and rounded shapes) by light microscopy (Figure 1A, B). By the 4^th^ week of culture, the adherent tumor cells display a more homogeneous morphology pattern in culture (Figure 1C). Cultured cells uniformly express CD44 in all three tumor samples (Figure 1D), but the labeling intensity is highly variable both between and within the same sample. Thus, compared to cells labeled with secondary antibody alone, the samples are 96%, 99% and 98% positive for CD44; however, the Mean Fluorescence Intensities (MFIs) of CD44 labeling are 10861, 5295 and 2120 respectively. Thus, the surface labeling intensity of CD44 expression may vary from 2 to 5 fold among tumor samples, and there is typically a large variance in average surface CD44 labeling *within* individual samples.

### Absence of Morphological Differences between CD44^hi^ and CD44^lo^ Cells

MPE-primary cultures acquire a more homogenous morphological pattern of growth over time. To determine if subtle differences in culture morphology could distinguish the CD44^hi^ from CD44^lo^ cultures, the M-1, M-2 and M-3 samples were labeled with anti-CD44 antibody and sorted by FACS, with gates set at 5% of cells at the high CD44 marker and low CD44 marker expression (Fig. 1E). The purity of the CD44^hi^ and CD44^lo^ cells were ≥98%, as revealed by post sort analysis (data not shown). The sorted cells CD44^hi^ (Figure 1F) and CD44^lo^ (Figure 1G) were washed and plated out in PCM for 2–3 days to evaluate their morphological differences. These studies suggest that there is no distinguishing difference in culture morphology associated with surface CD44 expression.

### CD44^hi^ Cells show High Colony Forming Ability

**Figure 2:**
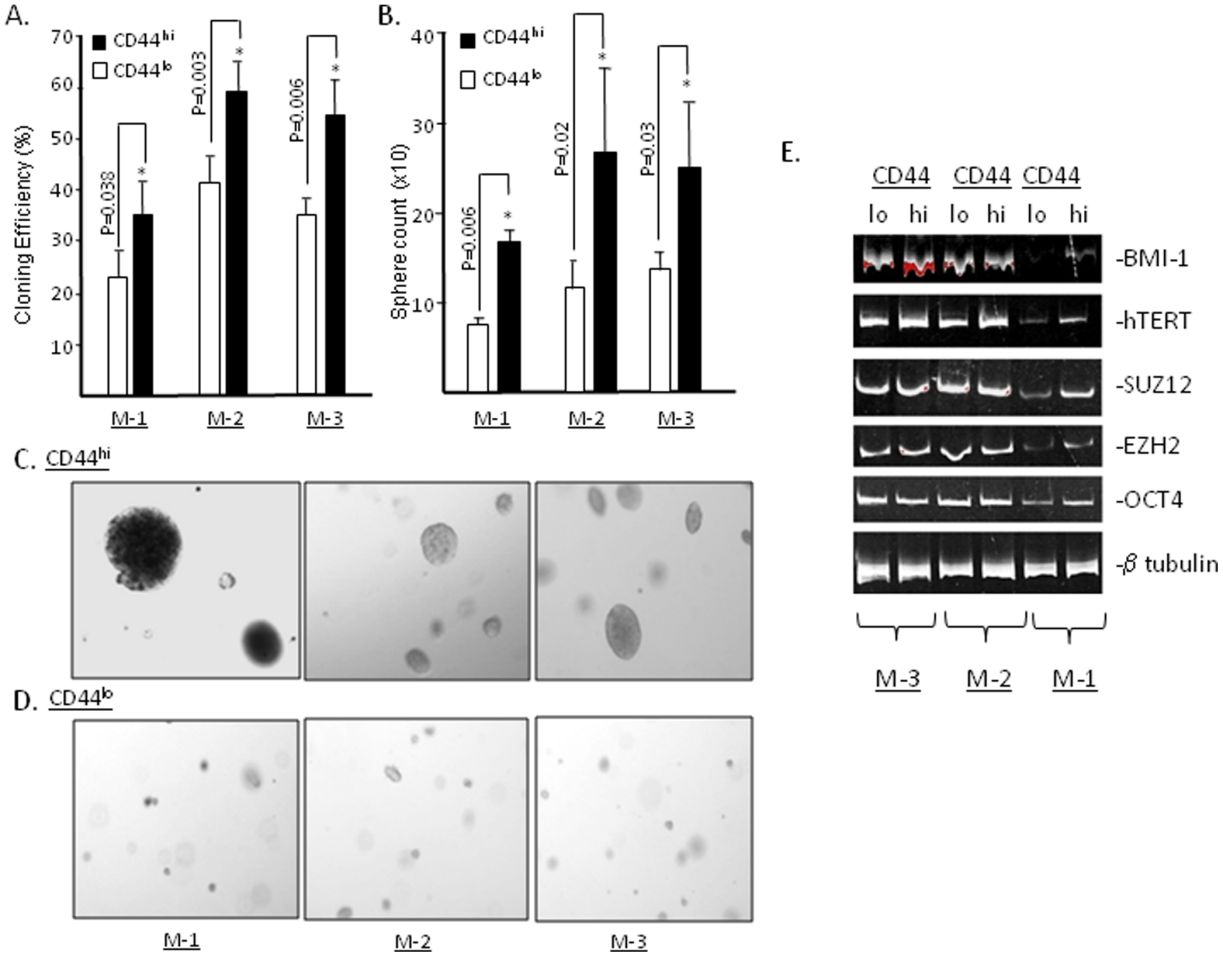
Higher clonal efficiency and colony forming potential of CD44^hi^ cells and their CSC molecular markers expression in comparison to CD44^lo^ cells. The sorted cells CD44^hi^ and CD44^lo^ from MPE samples were analyzed in triplicates for their (**A**) clonal efficiency (Sample M-1: colonies CD44^hi^ = 35.8 (SD = 5.04) vs CD44^lo^ = 21.7 (SD = 6.2) (P = 0.03); Sample M-2 colonies CD44^hi^ = 59.8 (SD = 3.2) vs CD44^lo^ = 40.6 (SD = 4.1) (P = 0.003); Sample M-3 colonies CD44^hi^ = 53.4 (SD = 5.3) vs CD44^lo^ = 33.9 (SD = 3.6) (P = 0.006)); The mean effect of CD44^hi^ versus CD44^lo^ is 17.6 (95% CI: 8.31, 26.89: p = 0.015). (**B**) colony forming ability in soft agar. (Sample M-1: colonies CD44^hi^ = 16.6 (SD = 1.1) vs CD44^lo^ = 8 (SD = 1.1) (P = 0.0006); Sample M-2: colonies CD44^hi^ = 27 (SD = 7) vs CD44^lo^ = 12 (SD = 3) (P = 0.02); Sample M-3: colonies CD44^hi^ = 24.3 (SD = 6.1) vs CD44^lo^ = 12.6 (SD = 2.5) (P = 0.03)). The mean effect of CD44^hi^ versus CD44^lo^ is 11.8 (95% CI: 3.41, 20.14; P = 0.026). Columns, mean from three independent experiment; SD, *, *P*<0.001, compared with the CD44^lo^ groups, (student’s *t* test). (**C**) Soft agar colonies derived from CD44^hi^ cells (100×) and (**D**) CD44^lo^ cells (100×). (**E**) Expression profile of BMI-1, hTERT, SUZ12, EZH2 and OCT-4 in sorted CD44^hi^ and CD44^lo^ cells were analyzed by reverse transcriptase-qPCR. In sample M-3 only BMI-1 is expressed at high level in CD44^hi^ population than the CD44^lo^ cell population. In sample M-2 slight higher expression of hTERT in CD44^hi^ cells than CD44^lo^ cells. The CD44^hi^ population of sample M-1 expressed high level of BMI-1, hTERT, SUZ12, EZH2 and OCT-4, than CD44^lo^ population.

To investigate whether the CD44^hi^ cells are functionally different from the CD44^lo^ cells in colony forming efficiency, we sorted and cultured these subsets from the three samples (M-1, M-2 and M-3). 100–500 cells of CD44^hi^ or CD44^lo^ cells were plated in individual wells of 12-well plates. Although we are unable to detect significant differences in initial plating efficiency, but we do observe that CD44^hi^ cells are more competent at forming holoclones than the CD44^lo^ cells (*t* test and ANOVA: P<0.05) (Figure 2A). Thus, an intrinsic biological difference between CD44^hi^ and CD44^lo^ cells seems to an inherent differential competency in forming holoclones.

### CD44^hi^ Cells show High Spheroid Forming Ability in Soft Agar Cultures

Another surrogate measure commonly used to characterize CSC is a differential competency at forming “anchorage independent” colonies in soft agar [12]. CD44^hi^ and CD44^lo^ cells from samples (M-A-1, M-10-26 and M-8-15) were evaluated by plating the sorted cells in agarose supplemented with PCM. The CD44^hi^ cells from all three samples uniformly exhibit higher spheroid formation efficiency than the CD44^lo^ cells (*t* test and ANOVA: P<0.05) (Figure 2B). The more robust CD44^hi^ colonies are also qualitatively distinguishable from vestigial colonies formed by CD44^lo^ cells (Figure 2C versus 2D). Thus, CD44^hi^ cells possess greater competency at forming colonies in soft agar than the CD44^lo^ cells derived from the same lung cancer biospecimen.

### CD44^hi^ Cells Variably Display Molecular Features that Characterize CSC

Markers that characterize *candidate* CSC (hTERT, SUZ12, OCT-4 expression etc.) are evident in cell pellets isolated from the MPE samples [5]; thus, CSC are a likely component of the MPE-tumor mix. Such CSC markers are variably comprised of embryonal or polycomb protein components and their expression may predict the labeling of cell subsets that possess high tumorigenic or colony forming potentials [27]. To determine if these *candidate* CSC markers were limited to specific CD44 sorted subsets, we screened the CD44^hi^ and CD44^lo^ cell subsets for differential mRNA expression; RT-PCR amplification of BMI-1, hTERT, SUZ-12, EZH2 and OCT4 was performed. Indexed to beta-tubulin mRNA, there is a marked variability in the expression of these markers within the CD44-sorted cell subsets (Figure 2E). For example, BMI-1 and hTERT mRNA is more highly expressed in CD44^hi^ cells than the CD44^lo^ cells in sample M-3 and M-2 respectively. Only in sample M-1, expected distributions of CSC markers (high BMI, hTERT, SUZ12, EZH2 and OCT-4) are evident in CD44^hi^ cells than the CD44^lo^ cells.

These results indicate that 1) molecular markers that encode for modifiers of chromatin structure or embryonal genes may be present in both highly tumorigenic and non-tumorigenic subsets of individual lung cancer cell populations, and 2) that there is marked variability in the differential expression of these candidate “CSC-biomarkers” in lung cancer biospecimens.

### CD44^hi^ Cells form Tumors in NOD/SCID (IL2rγ^null^) Mice

As specific molecular markers cannot reliably differentiate tumorigenic from non-tumorigenic cell subsets, can we distinguish these subsets on the basis of behavioral phenotypes? The CD44^hi^ and CD44^lo^ cell subsets from individual tumor cell populations consistently display differences in adherent holoclone and soft agar colony formation. A key experimental measure of “CSC”, however, is by the demonstration of higher tumorigenic potential in mouse models. It has been shown that NOD/SCID(IL2rγ^null^ mice are sensitive model to evaluate for highly tumorigenic CSC- behavioral phenotypes [16], [28]. To corroborate observed differences in colony forming and spheroid forming abilities of CD44^hi^ vs CD44^lo^ cells with *in vivo* tumorigenesis, we investigated their ability to form tumors in NOD/SCID (IL2rγ^null^) mice.

**Figure 3:**
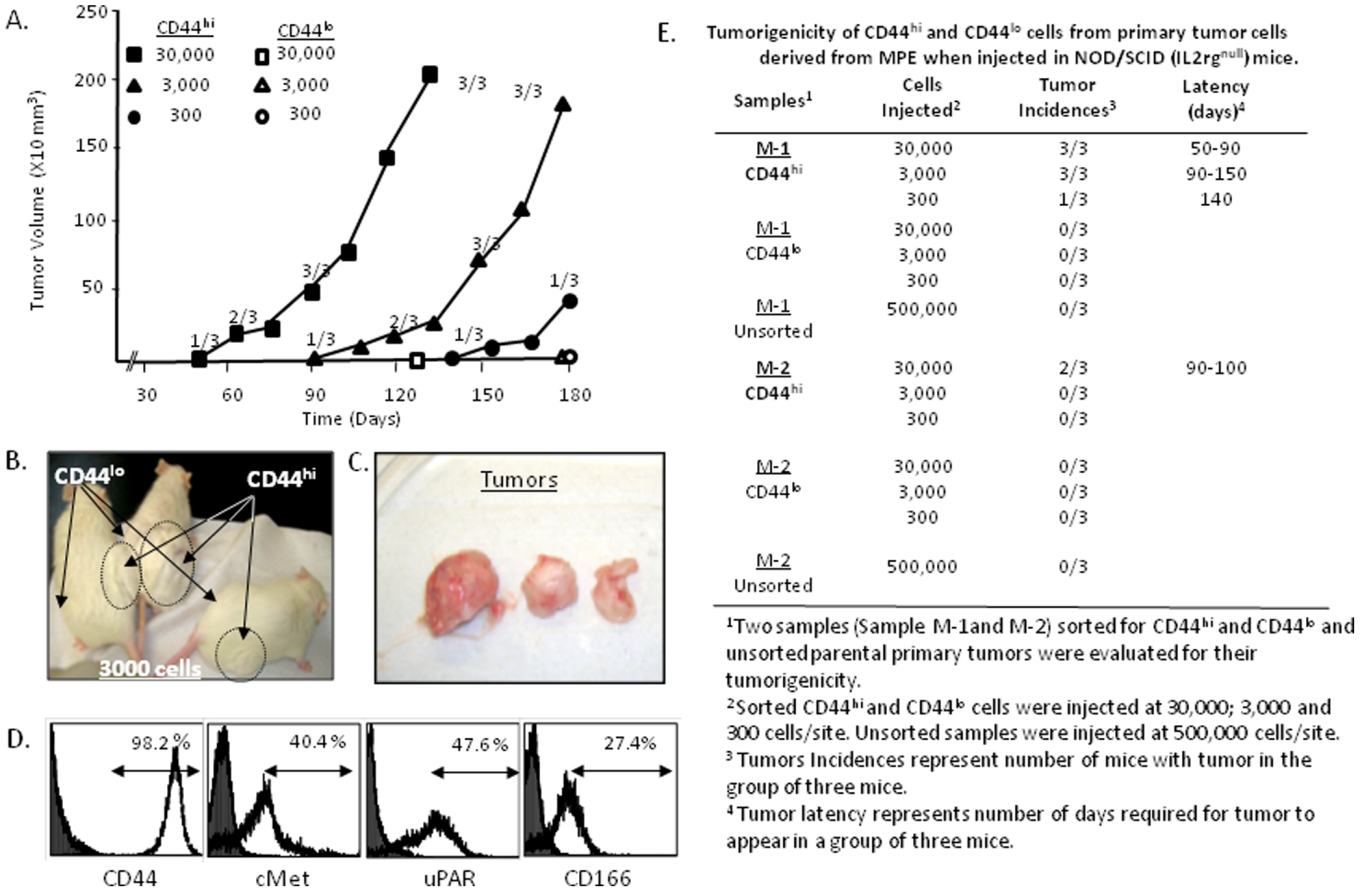
Tumorigenicity of CD44^hi^ population from primary tumor cells in NOD/SCID (IL2rγ^null^) mice. (**A**) Tumorigenicity and latency period of CD44^hi^ cells (M–1) injected with at 30,000; 3,000 and 300 cells. Mice injected with CD44^hi^ (right flank) formed tumors and CD44^lo^ cells did not form tumor (left flank). The numbers (1/3, 2/3 or 3/3) represent number of animals with tumor/group at particular time point of measurement. Time period of days after tumor implantation is expressed along X axis and tumor growth volume is expressed as mm^3^ along Y axis. Unsorted parental primary tumors implanted with 500,000 cell/mice did not show any tumor growth even after 3 months of tumor cell implantation (data not shown) (**B**) Mice bearing tumors on the right flank injected with CD44^hi^ cells and no tumor was detected at the left flank injected with CD44^lo^ cells (**C**) Resected tumors formed by CD44^hi^ cells in mice. (**D**) FACS analysis of single cells obtained from mouse tumors derived from CD44^hi^ cells of M-1 MPE sample. The tumor cells show expression of CD44, cMET, uPAR and CD166 markers. (**E**) Tumorigenicity and latency period of CD44^hi^ and CD44^lo^ cells of samples M-1 and M-2 in NOD/SCID (IL2rγ^null^) mice.

Limiting dilutions (30,000; 3,000; 300) of sorted CD44^hi^ and CD44^lo^ cells from M-1 and M-2 MPEs were injected into the right and left flanks respectively of NOD/SCID (IL2rγ^null^) mice. CD44^hi^ tumor cells of the M-1 sample formed tumors in 3/3 mice at both 30,000 and 3,000 injected cell doses, and in one of 3 mice injected with 300 tumor cells (Figure 3 A, B, C). The latency period of tumors was 50–90 days, 90–150 days and 150 days, for 30,000; 3,000 and 300 CD44^hi^ cells respectively (Figure 3E). Thus, the kinetics of tumor formation by the highly tumorigenic CD44^hi^ cells was dose-dependent. The CD44^hi^ tumor cells from sample M-2 generated tumors in 2 of 3 mice at 30,000 tumor cells with a latency period of 90–100 days, a higher latency period than observed in CD44^hi^ cells of sample M-1 (Figure 3E). Thus, although CD44^hi^ cells consistently display higher tumorigenic potentials than CD44^lo^ cells of the same specimen, individual tumor specimens may display different growth kinetics in the evaluation of CSC properties in behavioral bioassays.

Notably, the CD44^lo^ cells from either primary culture did ***not*** form tumors in the left flanks of the mice during the entire monitoring interval (Fig. 3 B and E). Moreover, we also did ***not*** observe tumor formation with the injection of 5×10^5^ *unsorted* cells, even though this population presumably contained ∼5–10% (or 25,000–50,000) CD44^hi^ cells. This interesting observation suggests that CD44^hi^ cells may be exposed to inhibitory influences towards tumor growth by cells that have a lower intensity surface CD44 expression in the same tumor population.

To test whether implanted CD44^hi^ cells contributed to heterogeneous tumors (suggestive of multipotent differentiation), engrafted tumors generated from CD44^hi^ from M-1 cells were extirpated, digested, and cell surface marker analysis was performed by FACS on single cell suspensions. The tumor cells remained highly positive for the CD44 marker, with 98.2% of cells staining positive, although the CD44-MFI was even higher than the originally implanted cells. Heterogeneity amongst cells was evidenced by the variable expression of other commonly associated CSC biomarkers (Figure 3D), [cMET (40.4%), uPAR (47.6%) and CD166 (27.4%)]. Cells bearing these markers were also previously detected in the primary MPE biospecimen samples, at varying fractions [5]. Together, these results indicate that CD44^hi^ cells derived from MPE are not only more tumorigenic than the CD44^lo^ cells, but that the CD44^hi^ cells are also capable of generating tumors with heterogeneous marker profiles, similar to those found in the primary MPE samples.

### CD44 and ALDH Expression in Implanted Xenografts Resemble Expression of these Markers in Archived Human Lung Cancer Pathology Specimens

**Figure 4:**
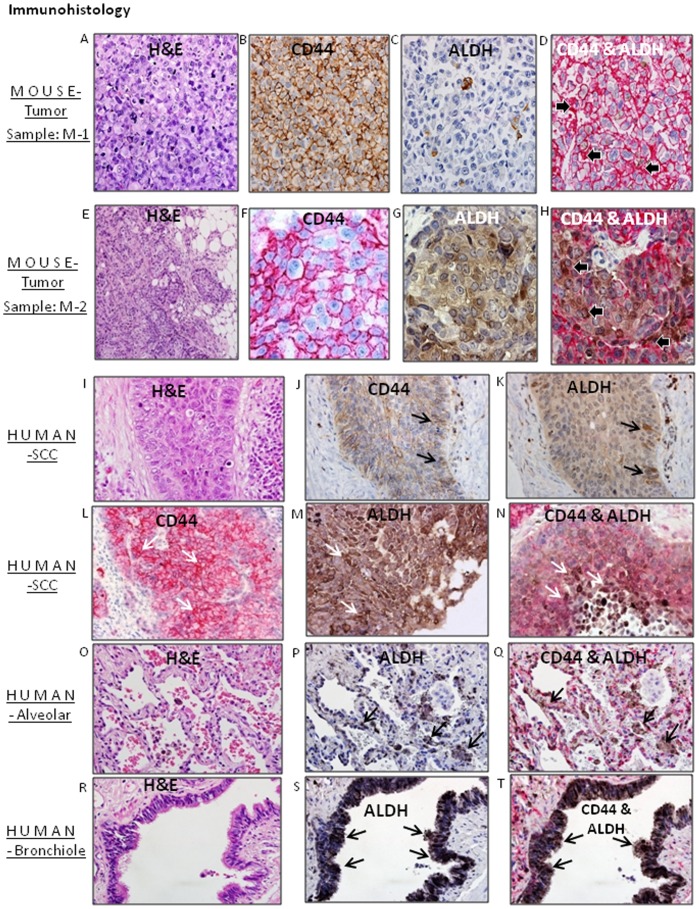
Immunohistological study of tumors generated by CD44^hi^ cell population in mouse, human squamous cell carcinomas (SCCs), human alveolar and human bronchiolar tissues. The photomicrograph A, B, C and D represent the tumors derived from Sample M-1 and E, F, G and H represent tumor derived from sample M-2 in NOD/SCID (IL2rγ^null^) mice. The following stains are represented: H&E staining (A and E), immunohistochemistry for CD44 expression (B and F), ALDH expression pattern (C and G) and the immunohistochemistry for dual CD44 and ALDH staining (D and H). Representative human two lung squamous cell carcinoma (SCC) tissue samples (I, J, K and L, M, N) were stained by H&E (I), CD44 (J and L), ALDH (K and M) and immunohistochemical staining for dual markers CD44 and ALDH (N). Human alveolar tissue sections were stained for CD44 (O), ALDH (P) and dual markers CD44 and ALDH (Q). Human-bronchiolar tissue sample was stained for CD44(R), ALDH (S) and dual markers CD44 and ALDH (T).

CD44^hi^ cells in MPE primary cultures contain cell fractions with high ALDH expression (i.e., the CD44^hi^/ALDH^hi^ surface phenotype) [5]. We extend that observation to prospectively collected biospecimens. Using immunohistochemistry, we observe variable expression of CD44 and ALDH markers in the mouse xenograft tumors generated from CD44^hi^ cells. Pathological and marker expression patterns in xenografts compare favorably to archived human lung cancer, and to tumor-adjacent human normal alveolar and normal human bronchiolar tissues. H&E sections of M-1 and M-2 CD44^hi^ xenografts (Figure 4 A–H) corroborate the original pathological diagnoses of large cell lung cancer and lung SCC respectively (Figure 4 I–N). Consistent with flow cytometry data, CD44 labeling is evident on the majority of cells. However, *intra*-tumoral variation of CD44 expression is clearly evident (Figure 4 B, F), again consistent with the flow cytometry profile. Similarly when the xenograft sections are labeled for ALDH expression, some cells show higher expression than other tumor cell populations (Figure 4 C, G). When co-expression of CD44 and ALDH is examined by dual marker staining of xenograft tumor sections, there is tumor to tumor variability in the co-localization of these markers (Figure 4 D, H).

These labeling patterns are representative of resected human pathology specimens, as evidenced by the morphology and immunohistopathology expression patterns observed in archived samples (Figure 4 I–J and L–M), which show CD44 (Figure 4 J, L), ALDH (Figure 4 K, M) and dual staining expression pattern of CD44 and ALDH (Figure 4 N). Arrows in the figure point to high expression of respective markers in SCC tissue sections (Figure 4 J–M). Dual expression of CD44 and ALDH in SCC tissue sections may be more intense toward the center of tumor nodules (Figure 4 N).

To determine if such labeling was restricted to the neoplastic tissue, tumor adjacent normal lung alveolar (Figure 4 O–Q) and bronchiolar (Figure 4 R–T) tissues were also examined. The photomicrographs suggest that high expression of ALDH (Figure 4 P), and co-expression ALDH and CD44 (Figure 4 Q) is evident in histologically normal alveolar tissues as well, where representative H& E photomicrographs of normal bronchiolar tissue shows the characteristic presence of ciliated and goblet cells (Figure 4 R). Foci of ciliated and goblet cells highly express CD44 or both CD44 and ALDH in this anatomical location (Figure 4 S, T). Since CD44 and ALDH markers are also co- expressed in normal lung tissues, the presence of these markers *per se* may not distinguish neoplastic from non-neoplastic tissues.

To determine if we could identify a relationship between CD44/ALDH expression and histopathological subtypes of lung cancer, tissue sections were evaluated for CD44, ALDH and co expression of CD44 and ALDH. CD44 and ALDH are commonly expressed in all lung tumor samples, both with respect to fractions of cell labeling, and intensity of labeling **(** **Table 1** **)**. The data suggest that SCC express higher levels (4+/3+) of CD44 and ALDH than adenocarcinoma, and co localization of these markers (the CD44^hi^/ALDH^hi^ surface phenotype) is also easier to identify in SCC (n = 5/7) than in adenocarcinoma (n = 1/12).

**Table 1:** CD44 and ALDH expression pattern in Squamous Cell Carcinoma (SCC) and Adenocarcinoma (AC) of the lung.

Case	Tumor Type	Grade	CD44 Staining		ALDH Staining		Co-localization
			Amount	Intensity	Amount	Intensity	
S-46	SCC	3+	4+	3+	1+	1+	Y
S-44	SCC	2+	1+	3+	2+	2+	Y
1–57	SCC	3+	4+	3+	3+	2+	Y
S-24	SCC	3+	4+	3+	4+	3+	Y
S-34	SCC	3+	4+	3+	1+	1+	Y
1-2T	SCC	3+	0+	0+	1+	2+	NA
1–76	SCC	2+	4+	3+	0+	0+	NA
9–69	AC	3+	0+	0+	0+	0+	NA
1–68	AC	2+	0+	0+	0+	0+	NA
8–94	AC	3+	1+	3+	0+	0+	NA
S-06	AC	1+	4+	3+	0+	0+	NA
1–70	AC	2+	0+	0+	2+	2+	NA
1-2T	AC	1+	0+	0+	0+	0+	NA
1-08	AC	3+	0+	0+	3+	3+	NA
S-57	AC	2+	2+	3+	0+	0+	NA
S-42	AC	1+	0+	0+	0+	0+	NA
1–18	AC	3+	0+	0+	0+	0+	NA
9–79	AC	2+	1+	2+	2+	2+	N
S-35	AC	1+	4+	3+	1+	1+	Y

**Tumor Type**

SCC = Squamous cell carcinoma

AC = Adenocarcinoma.

**Grade**

1+ = Well differentiated

2+ = Moderately differentiated

3+ = Poorly differentiated.

**Amount of Staining**

0+ = <5% of cells

1+  = 6 to 24% of cells

2+  = 25 to 49% of cells

3+  = 50 to 74% of cells

4+  = 75% or greater of cells.

**Intensity of Staining**

0+ = no staining

1+ = weekly positive

2+ = moderately positive

3+ = strongly positive.

**Co-localization**

N = No co-localization

Y = co-localization identified

NA = Not Applicable.

### Rearrangement of Chromosome 1p36 and Reduced Expression of miR-34a in CD44^hi^ Cells

**Figure 5:**
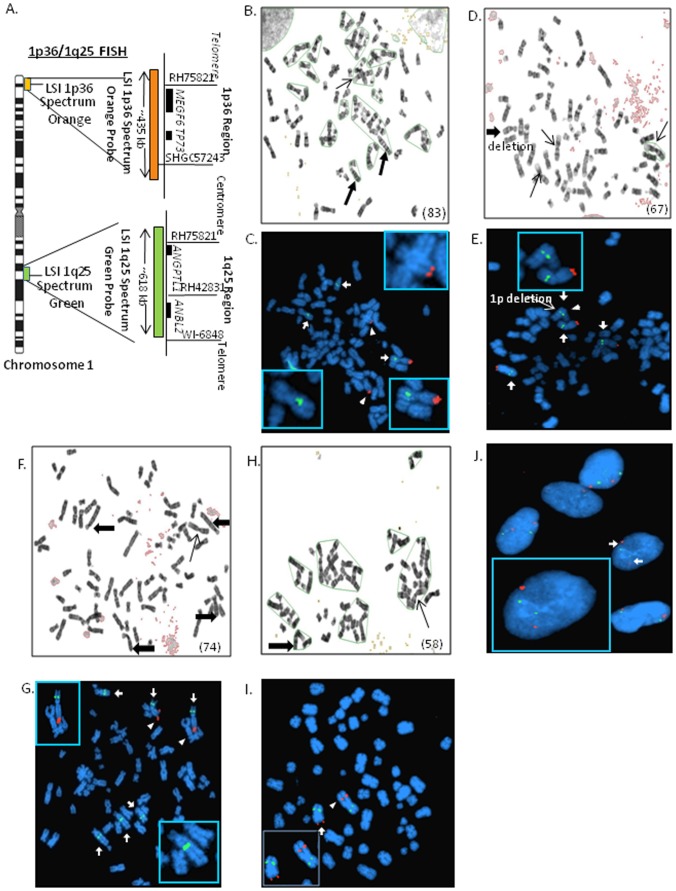
Karyotype and Fluroscent In Situ Hybridization (FISH) analysis of MPE derived tumor cells. (**A**) Dual color FISH analysis was done using 1p36 and 1q25 (control) probes. Representative position of the probe 1p36 (orange) and 1q25 (green) on chromosome 1. (**B**) Sample M-1: Abnormal hyperdiploid karyotype (83 chromosomes) and (**C**) with 3 copies of chromosome 1 (**↑**) but have 2 copies (**Δ**) of rearranged 1p and 1q. (**D**) Sample M-2: Abnormal hyperdiploid karyotype (67 chromosomes) and (**E**) with 4 Chromsome 1 s (**↑**) (3 with intact 1p/1q and 1 with 1p deletion (**Δ**)). (**F**) Sample M-3: Abnormal hyperdiploid karyotype (74 chromosomes) and (**G**) with 2 copies of 1p (**Δ**) and 6 (**↑**) copies of 1q (consistent with 1p deletion). (**H**) Sample NCI-H2122 Abnormal karyotype (58 chromosomes) and (I) with 2 copies (**↑**and **Δ**) of 1p/1q but one 1p is rearranged with additional material of unknown origin at 1p terminal region (**Δ**). (J) Normal deployed human fibroblast cell line GM 05399 control with two copies of 1p/1q (**↑**).

Abnormal chromosomal numbers, and both hyper- and aneuploidy are common in lung cancer. It is not clear whether such chromosomal changes are associated with the tumorigenic potential of cancer cells. To investigate a possible association, karyotype analysis was performed on the three MPE samples. Normal fibroblast GM 05399 and the lung cancer cell line NCI-H2122 were used as controls to represent non tumorigenic and immortalized tumor cell models [20]–[23]. All three MPE samples M-1, M-2 and M-3 showed extensive chromosomal changes with hyperdiploid number of chromosomes 83, 67, and 74 respectively (Figure 5B, D, F). Meanwhile, the normal fibroblast contained 46 chromosomes; the cell line NCI-H2122 contained 58 chromosomes (Figure 5H). MPE cells uniformly contained translocations and deletions, and rearrangements at chromosomal region 1p, a common site of rearrangements seen in lung cancers. A FISH analysis was carried out using a 1p36 (orange) probe and a control 1q25 (green) probe to detect specific 1p changes (Figure 5A). Sample M-1 has 3 copies of chromosome 1 (**↑**), of which 2 copies (**Δ**) are rearranged at 1p and 1q (Figure 5C). The sample M-2 exhibits 4 Chromosome 1 (**↑**) (3 with intact 1p/1q and 1 with 1p deletion (**Δ**)) (Figure 5 E). The third sample M-3 has 2 copies of 1p (**Δ**) and 6 (**↑**) copies of 1q (consistent with 1p deletion) (Figure 5E).

The immortalized MPE-derived lung cancer cell line (NCI-H2122) also displays an abnormal karyotype with hyperploidy (Figure 5H). NCI-H2122 has 2 copies (**↑**and **Δ**) of 1p/1q but one 1p is rearranged with additional material of unknown origin at 1p terminal region (**Δ**) (Figure 5I). By contrast, the normal diploid human fibroblast GM 05399 cells show normal distribution of two copies of 1p/1q (**↑**) (Figure 5J).

Thus, we detected Loss of Heterozygosity (LOH) at 1p36 in two MPE samples and rearrangements of both 1p/1q regions in the third MPE sample. Cell line H-2122 contained one normal chromosome 1 and unbalanced translocation of unknown origin at 1p36 consistent with deletion of 1p. The observations suggested that 1p36 deletion could result in the inactivation of a tumor suppressor gene. A bioinformatics search identified candidates, including the code for miR-34a that mapped to this locus.

**Figure 6:**
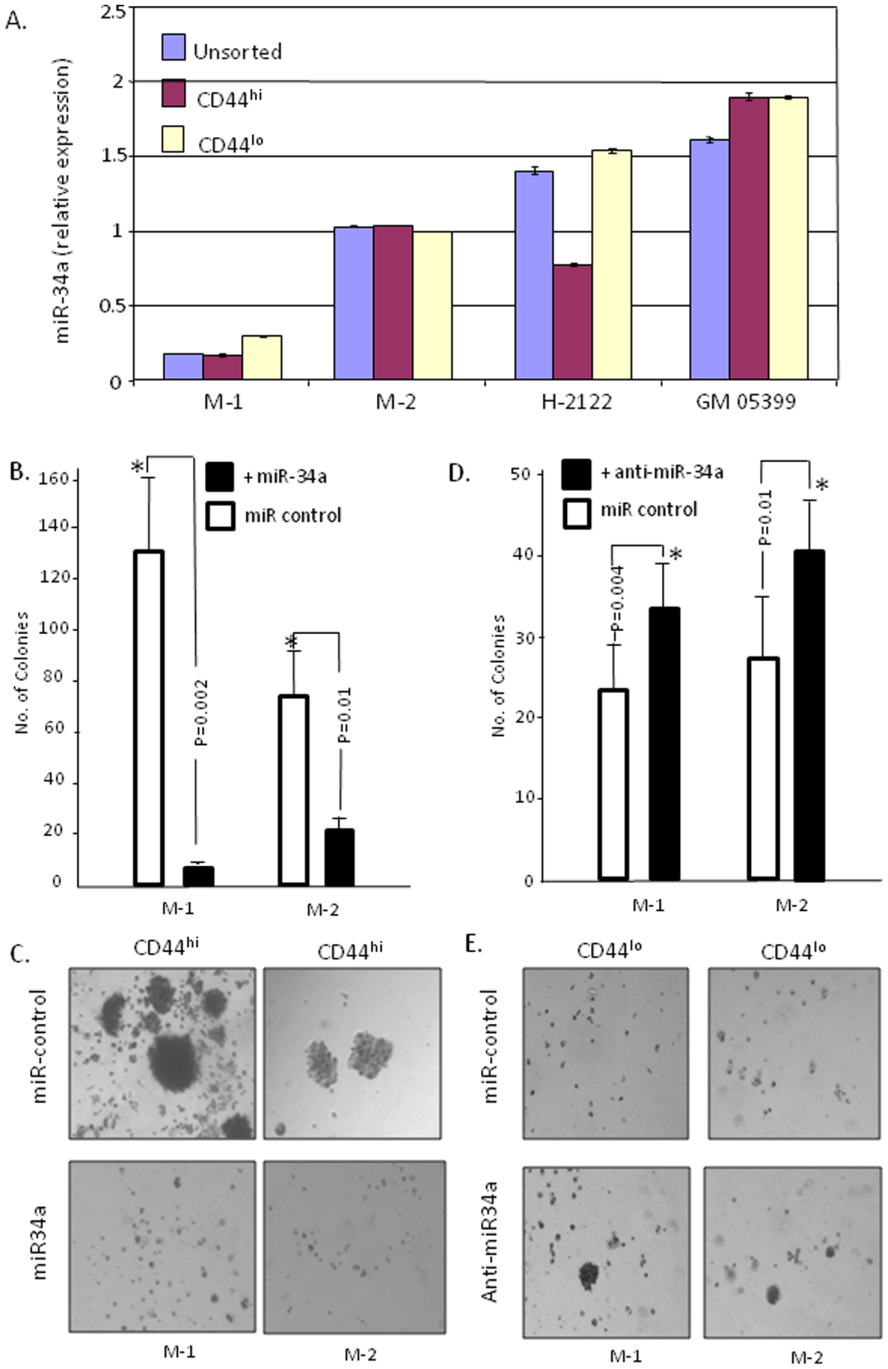
Expression of miR-34a in CD44^hi^ and CD44^lo^ cells evaluated by RT-qPCR and exogenous delivery of miR-34a into CD44^hi^ cells inhibits colony formation and anti- miR-34a into CD44^lo^ cells increases colony formation. (**A**) miR-34a expression in unsorted, CD44^hi^ and CD44^lo^ cells of two primary samples (M-1 and M-2), established NSCLC cell line NCI-H-2122 and normal human fibroblast cell line GM 05399. The miR-34a expression has been normalized with RNU48. (**B**) Sorted CD44^hi^ cells (samples M-1 and M-2) when transduced with miR-34a show decreased colony forming efficiency in comparison to miR-control transduced CD44^hi^ tumor cells; (Sample M-1: miR control = 131.6 (SD = 30.5),+miR-34a = 7 (SD = 2.6) and P = 0.002; Sample M-2: miR control = 75 (SD = 19.6),+miR-34a = 23 (SD = 5.5) and P = 0.01); The mean effect with miR-34a versus miR-control on CD44^hi^ cells is −88.3 (95% CI: −288.12, 111.45; P = 0.112) (**C**) Sorted CD44^hi^ tumor cells transduced with miR-34a exhibit small colony size (bottom panel) than miR-control transduced CD44^hi^ tumor cells (upper panel); (**D**) The CD44^lo^ cells from Sample M-1 and M-2 transduced with anti-miR34a show increased colony forming efficiency than tumor cells transduced with miR-control; (Sample M-1: miR control = 21 (SD = 4.5),+anti- miR-34a = 33 (SD = 5.2) and P = 0.04; Sample M-2: miR control = 24.6 (SD = 4.1),+anti-miR-34a = 39.3 (SD = 5.1) and P = 0.018); The mean effect with anti-miR-34a versus miR-control on CD44^lo^ cells is −13.3 (95% CI: −20.43, 47.10; P = 0.125). (**E**) Sorted CD44^lo^ cells transduced with anti-miR-34a exhibit bigger colony size (bottom panel) than miR-control transduced tumor cells (upper panel).

Since expression of miR-34a may contribute to the different biological properties of CD44^hi^ versus CD44^lo^ cells in individual tumors, we evaluated its expression levels in CD44^hi^, CD44^lo^ and unsorted total cell populations in fractionated MPE-biospecimens (Figure 6A). The expression of small nucleolar RNA-RNU48 was used as a reference for gene expression in this assay (data not shown), and miR-34a results were normalized with the RNU48 expression. Although the expression of the small nucleolar RNA-RNU48 may itself be dysregulated in cancer [29], our study demonstrated a similar basal expression pattern across the sample sets. On RT-qPCR analyses, however, there was no significant difference in miR-34a expression in the CD44^hi^ and CD44^lo^ subsets of the MPE sample M-2; the expression in this sample was similar to that of the control fibroblasts. In contrast, CD44^hi^ cells have significantly lower level of miR-34a than the CD44^lo^ cells in sample M-1, as well as in the immortalized cell line NCI-H2122. These data suggest that loss of miR34a may contribute to aggressive biological properties and high tumorigenic potentials in some lung cancers.

### Soft Agar Colony Formation by CD44^hi^ Cells is Correlated with the Decreased Expression of miR- 34a in Individual Lung Cancers

To determine whether the decreased expression of miR-34a could be directly associated with an aggressive phenotype in some lung cancers, we compared colony forming ability and tumorigenic potentials of CD44^hi^ tumor cells with their miR-34a expression. The loss of miR-34a in these samples directly correlated with a competency at high colony formation. Thus, CD44^hi^ cells with the lowest miR-34a expression formed a higher number and larger colonies, while CD44^lo^ cells with higher miR-34a expression formed smaller number of vestigial colonies (Figure 2). Fibroblasts, with high miR-34a expression, failed to form colonies in soft agar (data not shown).

To further assess the role of miR-34a towards mediating a biological effect in tumor cells subsets, CD44^hi^ and CD44^lo^ cell populations were transfected with miR-34a or anti-miR-34a, and colony formation was assayed. Introduction of miR-34a into CD44^hi^ cells resulted in 80–95% reduction of soft agar colonies (*t* test: P = 0.01–0.002) (Figure 6B, C). As expected, introduction of anti-miR-34a into CD44^lo^ led to increased number of soft agar colonies (*t* test: P = 0.04–0.01) (Figure 6D, E). The results were significant by *t test* analysis as indicated in Figure 6B and 6D. Though the differences were significant, however, by ANOVA test the P values were 0.112 (Figure 6B) and 0.125 (Figure 6D), indicating that either the variability within the two samples were greater or the sample numbers were few to be significant by ANOVA analysis. However, miR-34a clearly plays an important role in tumor growth suppression; the loss of miR-34a expression is evident in aggressive (CD44^hi^ subsets) of individual cancers, and this loss directly contributes to the development of a highly tumorigenic phenotype.

### CD44^hi^ Cells Display Extended G2 Phase Cell Cycle

It is believed that CSCs remain in quiescent state and cycle slower through the cell cycle; these are properties resembling normal stem cells [30]. We evaluated the cell cycle phase of the CD44^hi^ cells that show higher tumorigenic potential by FACS.

**Figure 7:**
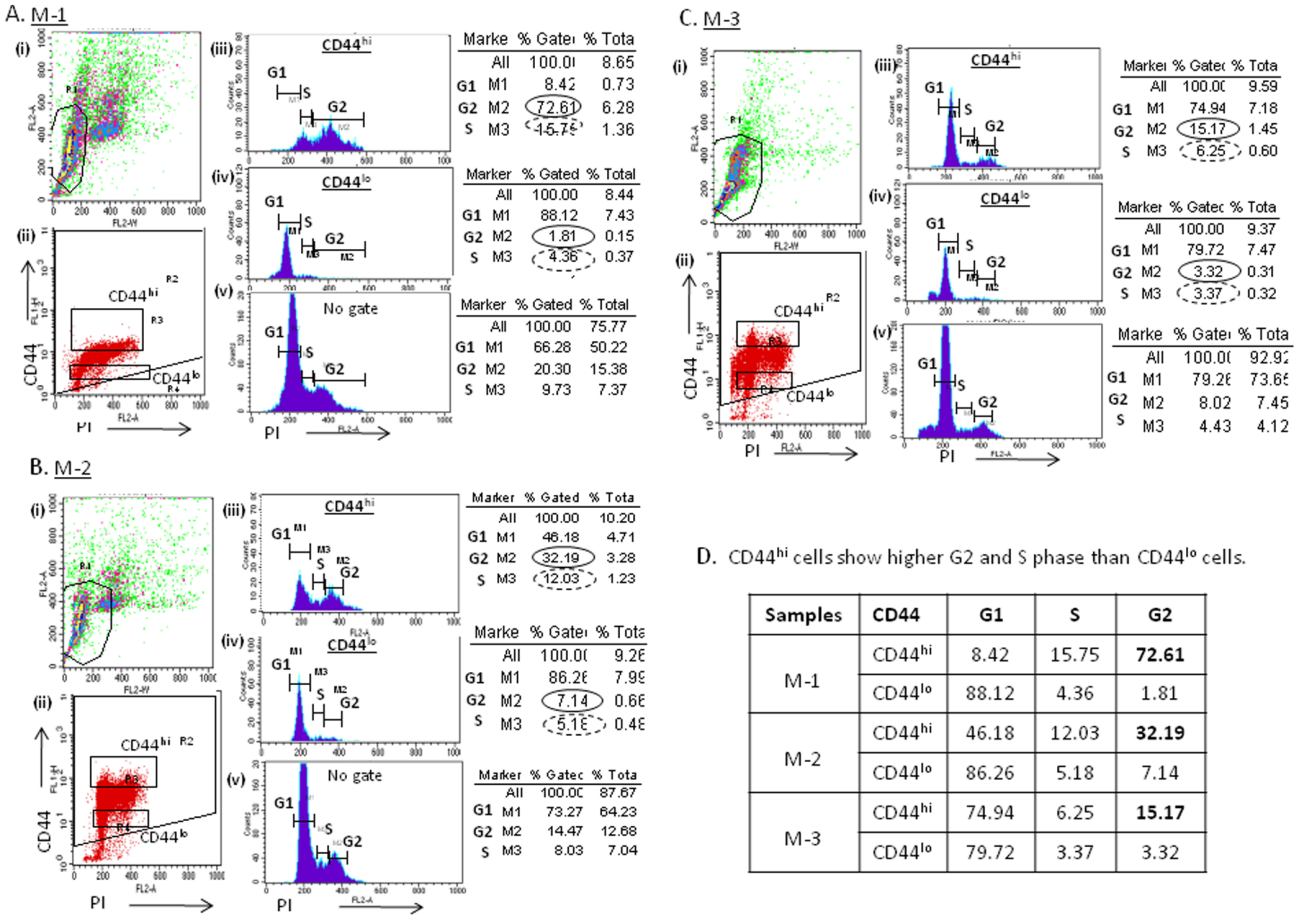
Cell cycle parameters of CD44^hi^ and CD44^lo^ cell populations derived from primary cultures of MPE tumors. Representative FACS analysis of CD44 and PI staining (i and ii) of three samples (**A**) M-1, (**B**) M-2 and (**C**) M-3. The samples were evaluated for their CD44^hi^ (iii) and CD44^lo^ (iv) and total cells (v, no gate) cell cycle and their G1, S and G2 phase analysis. (**D**) The results indicate that gated CD44^hi^ population express high level of S and G2 phase (Sample M-1: S/G2∶15.75/72.61; Sample M-2: S/G2∶12.03/32.19; Sample M-3: S/G2∶6.25/15.17) than the gated CD44^lo^ population (Sample M-1: S/G2∶4.36/1.81; Sample M-2: S/G2∶5.18/7.14; Sample M-3: S/G2∶3.37/3.32) respectively.

Samples (A) M-1, (B) M-2 and (C) M-3 were stained for CD44 and PI and then first gated with PI staining pattern (Figure 7i) and then back-gated for CD44 (CD44-FITC/FL-1) and PI (FL-2A) (Figure 7ii). The panels iii, iv and v of figure 7 represent histogram of cell cycle stages of CD44^hi^ and CD44^lo^ gated cells (5–10% of total cells) and un-gated total cell population respectively. Figure 7D represents the population at different cell cycle stages G1 (M1), G2 (M2) and S (M3) stages. The CD44^hi^ cells of sample (A) M-1 shows that S and G2 phases are 15.75% and 72.61% of the gated cell population respectively (Figure 7A iii). CD44^lo^ cells of the same sample represent 4.36% (S) and 1.81% (G2) of gated cell population (Figure 7A iv). Thus CD44^hi^ cells are enriched 40-fold for cells in the G2 phase than the CD44^lo^ cells.

Similarly, sample (B) M-2 analysis indicates that CD44^hi^ cells in S/G2 phase are higher (12.03/32.19) than the CD44^lo^ cells (S/G2∶5.18/7.14) (Figure 7B iii and iv and D). In this sample, the CD44^hi^ cells are enriched for cells in S/G2 phase at 2.3/4.5 times higher than CD44^lo^ cells. In the third sample (C), M-3, cells in S/G2 phase represent 6.25/15.17 percent of the whole population, where CD44^lo^ cells in the as S/G2 represent 3.37/3.32 respectively (Figure 7C ii and iii and D). The gated CD44^hi^ cells at S/G2 cell cycle stages are 1.8/4.5 times higher than gated CD44^lo^ cells. In all three samples, majority of CD44^lo^ cells reside at G1 phase of the cell cycle.

The data indicate that the CD44^hi^ cells are enriched for S and G2 phase fractions more than the CD44^lo^ cells indicating slow growth, quiescence of these cells.

## Discussion

Our previous study detected intratumoral heterogeneity in advance stage of lung cancer by surface marker analysis, immunohistochemistry (CD44, ALDH, cMET, MDRI) and FACS (CD44, ALDH, cMET, CD166, MDR-1, uPAR) [5]. This study extends the earlier observations, and also verifies that subsets of MPE tumor cells express variable levels of embryonal and polycomb complex-associated molecular markers. These stem cell markers have previously been implicated in mediating “CSC properties”, including high tumorigenic potentials. These markers include (but are clearly not limited to) PTEN, OCT-4, BMI-1, hTERT, SUZ12, EZH2. In early analyses, we are unable to associate specific embryonal or polycomb markers with higher tumorigenic potentials. In the three current MPE primary samples tested, only one of the CD44^hi^ subsets expressed (M-1) the predicted pattern of candidate CSC-marker expression (*lower* PTEN, *higher* hTERT, SUZ12, EZH2, OCT4 and BMI1) than the isogenic CD44^lo^ cells. The other two samples (M-2 and M-3) were quite variable in the expression of markers on this panel. On the basis of a primary samples (n = 3) that displays a highly variable expression of markers, we can speculate that it is unlikely that *individual* molecular markers will reliably predict the highly tumorigenic CSC-phenotype in lung cancers.

Whereas our earlier studies focused on demonstrating that candidate CSC existed in MPE by virtue of surrogate biomarker expression, this study actually associates the expression of those biomarkers with behavioral bioassays (colony formation and tumorigenesis *in vivo*). We clearly demonstrate that within the MPE-tumor biospecimen there are tumor cell subsets (CD44^hi^ cells) with high tumorigenic potentials. Thus, these subsets can now be characterized as having properties associated with “cancer stem cells” in three distinct surrogate measures of that property. Our data also suggest that lung CSC can be distinguished from non-CSC on the basis of several associated molecular properties and profiles. Although many additional properties are likely to emerge with prospective high throughput analyses, this report provides initial evidence of differences in cell cycle profiles, and in miRNA expression. Collectively, our studies convincingly demonstrate that behaviorally aggressive (CSC or tumor initiating cells) are present within the bulk MPE populations of lung cancer patients.

The CD44^hi^ cell subsets from different primary tumor cultures consistently formed tumors *in vivo* with greater efficiency (Figure 3). However, these efficiencies and tumor growth kinetics varied quite dramatically from one sample to another. The surface labeling intensity of CD44 indicated a better proxy marker for growth kinetics. The CD44^hi^ cells from the fast growing M-1 sample displayed higher surface CD44 (MFI = 28243), as compared to the CD44^hi^ cells from relatively slow growing M-2 sample (MFI = 12864) (Figure 1E). The CD44^hi^ cells from the M-1 tumor exhibited a more primitive phenotype (in terms of expected BMI, hTERT, SUZ12, EZH2, OCT-4 expression), as compared to the CD44^hi^ cells from the M-2 sample (with only higher hTERT expression) (Figure 2E). Thus, CD44^hi^ cells from the M-1 sample were much more efficient at forming *in vivo* tumors than the CD44^hi^ cells from M-2 sample. These data suggest that whereas the CD44^hi^ surface phenotype may commonly predict for more efficient tumorigenesis in individual tumors, there are likely to be differences in the molecular signatures that comprise this highly tumorigenic subset.

As indicated, the main objective of the present study was to identify and extract the tumor cell subpopulations from MPE that are responsible for tumor propagation and maintenance, and to characterize their molecular signature pattern. CD44 had previously been implicated as a surface marker for CSC as indicated earlier. Our earlier studies convincingly showed that almost all the MPE primary tumor cells labeled for surface CD44 (>98%). To distinguish a behaviorally-distinct cell subset amongst a cell population that contiguously expressed the CD44 surface marker, we elected to compare tumorigenic potentials of MPE-tumor cells expressing the highest levels of surface CD44 (CD44^hi^) with tumor cells expressing the lowest level of surface CD44 (CD44^lo^). It was not possible to distinguish these cell subsets simply on the basis of morphology; i.e.: cells sorted on the basis of CD44^hi^ and CD44^lo^ are morphologically similar. However, the CD44^hi^ cells could be clearly distinguished by behavioral properties, such as high clonal efficiency and high spheroid formation efficiency in soft agar, the established surrogate *in vitro* properties of CSC like cells. Accordingly, this study identifies the CD44^hi^ surface phenotype as a marker that is associated with high tumorigenic potentials in individual lung cancers. However, the surface phenotype may not be associated with a consistent molecular profile. More importantly, this study does not predict that the surface CD44^hi^ phenotype is *exclusively* the cancer cell subset with higher tumorigenic potentials. Clearly, the surface CD44^hi^ phenotype is not a homogeneous population. First, the expression of the CD44 surface marker varies greatly from one tumor to another. Moreover, surface CD44 expression varies greatly between individual tumors; the tumor cells that most highly label for surface CD44 seem to possess greater competence at tumor formation.

That the CD44^hi^ subset is not a homogeneous cell subset is suggested by the co-labeling of subsets with additional candidate CSC markers (e.g.: ALDH). Only a fraction of the CD44^hi^ subpopulation can be jointly characterized as the CD44^hi^/ALDH^hi^ surface phenotype [5]. In order to investigate if there is a co-relationship between CD44 and other known marker of CSC/TIC we evaluated one of the most prominent markers, ALDH, for its expression pattern by immuno-histpathology in the tissues generated by CD44^hi^ implanted cells in NSG mice and primary SCC and AC of lung cancer. It is suggested that various isozymes of ALDH are expressed in different lung cancer cell lines [31] and ALDH expression is significant for poor prognosis [32]. ALDH, like CD44, may also have a functional role in cancer progression [33], [34]. Our study has shown that only a small fraction of CD44^hi^ subpopulation can be jointly characterized as CD44^hi^/ALDH^hi^ surface phenotype in xenograft tissues and SCC and AC of the lung cancer.

Chromosomal abnormalities are common in cancer and in lung cancer losses and/or gains of several chromosomal regions have also been reported [35]. We were interested to evaluate if chromosomal abnormalities are also detected in the MPE samples, as has been reported for lung cancer. To evaluate these abnormalities we performed G-banded karyotype analysis and chromosome painting by using Fluorescence In Situ Hybridization (FISH). Our result indicated hyperploidy and chromosomal abnormality in all the MPE samples tested. FISH analysis of 1p36 region revealed LOH in two samples and rearrangements of both 1p/1q regions in the third MPE sample. Thus, indicating important role of region 1p36 in MPE where miR-34a maps. In this respect, data presented herein suggest that miR-34a likely represents a key etiologic factor in contributing to aggressive CSC phenotypes, and is thus a likely target for curbing the growth potentials of lung CSC in a subset of lung cancers. Specifically, a relative loss of miR-34a expression appears to contribute to aggressive behavioral features of lung CSC, and those features can be mitigated by exogenous delivery and restoration of miR34a activity.

Deletion of 1p36 in neuroblastoma has led to identification of a number of tumor suppressor genes from a 2 Mb region of this locus. These genes include TP73, CHD5, K1F1B, CAMTA1, and CASTOR [36]. The p53 induced miRNA-34a also localizes to this site, and is considered to be a strong candidate tumor suppressor gene in neurobalstoma and other human cancers. Studies have shown a suppressive effect on N-myc expression in neurobalstoma [36] and CD44 in prostate cancer [15], supporting a role in cancer suppression. In our system the MPE derived CD44^hi^ cells exhibited low expression of miR-34a.

Mir-34a has been also associated with regulation of cancer stem cells function in various cancer types such as prostate cancer [15], pancreatic cancer [37], meduloblastoma [38], glioblastoma [39]. Further, the microRNA miR-34a inhibits prostate cancer stem cells and metastasis by directly repressing CD44 indicating direct role of CD44 and mir-34a in cancer development and progression [15]. In lung cancers, miR-34a is being evaluated as a replacement therapy candidate; exogenous gene delivery of miR-34a can reduce tumor growth [40], [41].

CSC may maintain themselves at a particular stage of cell cycle due to differences in cell cycling or activation of checkpoints due to DNA damage. Recently, Harper et al., [30] showed that CD44^hi^ cells from both normal and malignant epithelial tissues have extended G2 cell cycle phase, which is associated with drug resistance. Our results suggest that even without the use of drug selection pressure, the CD44^hi^ subset is enriched for cells in the G2 phase of the cell cycle. The CD44^lo^ cells, by contrast, are enriched for cells in the G1 phase.

The three MPE samples evaluated represent the same stage of disease progression. However, each specimen is variable both in terms of histopathological subtype, and *grade* of differentiation. It is currently not known whether poorly differentiated cells are biologically more aggressive, but many have postulated this to be the case. The M-1 sample is from a younger patient and has more poorly differentiated cancer cells than sample M-2 and M-3. In our evaluation, the poorly differentiated cells in this sample were indeed more tumorigenic, however, this observation needs additional confirmation. Nevertheless, our pilot data suggests there is considerable intra-tumoral heterogeneity at an advanced stage of progression. In addition, despite a similar clinical stage of disease, there is considerable inter-tumoral heterogeneity between the clinically isolates, based on the fractional expression of individual markers and cytopathology. Although our examination is limited in its scope, these data suggest that understanding the biological and functional basis of this heterogeneity may enable us to better understand and develop rational therapeutics for lung cancer. We are actively seeking resources to expand this scope of study. However, it is important for us to point out that irrespective of the underlying histopathological subtype, CD44^hi^ cells are present in each biospecimen. Perhaps, this observation suggests that irrespective of the histopathological subtype, the genetic and epigenetic landscape of CD44^hi^ tumor cells may be similar across lung cancers. If this hypothesis holds to be true, then we may be in a position to offer common CD44-biomarker guided therapeutics across lung cancer subtypes.

In summary, this work substantiates the validity of our lung cancer MPE model and phenotype-based approach for the discovery of the molecular bases of functional intratumoral heterogeneity. This work extends the evidence to support our proposition that for us to effectively treat cancer, we need to approach the disease starting from a behavioral phenotype. The most efficient way for us to accomplish that task is to dissect the molecular basis of specific properties in behaviorally distinct cell subsets of individual tumors [42].

## Supporting Information

Supporting Information S1 **Cytopathology of sample M-1, M-2 and M-3.**
(DOC)

Supporting Information S2 **Malignant Pleural Effusion (MPE) collection, processing and cell culture.**
(DOC)
