# Correction: The CD44^high^ Tumorigenic Subsets in Lung Cancer Biospecimens Are Enriched for Low miR-34a Expression

**DOI:** 10.1371/annotation/9ae8db84-5d44-4a9c-bb80-47c9d68820ea

**Published:** 2013-09-30

**Authors:** Saroj K. Basak, Mysore S. Veena, Scott Oh, Chi Lai, Sitaram Vangala, David Elashoff, Michael C. Fishbein, Sanjai Sharma, Nagesh P. Rao, Dinesh Rao, Ryan Phan, Eri S. Srivatsan, Raj K. Batra

The following information disclosure by the author was omitted from the article: "Raj K. Batra is an inventor on a relevant patent application PCT/US2013/050712 entitled METHODS OF BIOMARKER VALIDATION AND TARGET DISCOVERY filed in July 2013.

This patent application does not alter the authors' adherence to all the Plos ONE policies on sharing data and materials. Therefore all materials presented in the manuscript will be freely available to the scientific community although not for commercial purposes.” 

