# Supplementary material for: The CD44high Tumorigenic Subsets in Lung Cancer Biospecimens Are Enriched for Low miR-34a Expression
Source: PLoS One. 2013 Sep 3;8(9):e73195. doi: 10.1371/journal.pone.0073195 (PMC3760902; doi:10.1371/journal.pone.0073195)
Supplement: Supporting Information S1 — Cytopathology of sample M-1, M-2 and M-3. (DOC) [file pone.0073195.s001.doc]

**Supplemental Information:**

**S-1:** *Cytopathology of sample M-1, M-2 and M-3.* Subjects presenting with suspected malignant pleural effusions underwent informed consent by an IRB-approved protocol at VAGLAHS. Large volume thoracentesis were performed for diagnostic and/or therapeutic purposes.  Aliquots were separated for diagnostic cytopathology and primary culture (as described in Ref. 5, Basak et. al. PLoS, 2009). The diagnostic cytopathology is depicted, alongside cell counts and differential analyses of non-tumor cell populations.

|  | M-1 | M-2 | M-3 |
| --- | --- | --- | --- |
| Age | 50 | 62 | 73 |
| Gender | M | M | M |
| Ethnicity | African American | White | African American |
| Cytopathology | poorly differentiated non-small cell carcinoma | Non-small cell carcinoma, adenocarcinoma | Non-small cell carcinoma |
| RBC/uL | 240,000 | 430,000 | 400,000 |
| WBC/uL | 1050 | 300 | 550 |
| Neutrophil % | 79 | 6 | 36 |
| Lymphocytes% | 17 | 43 | 50 |
| Monocytes% | 3 | 1 | 10 |
| Eosinophils% | 1 | 39 | 4 |
| Mesothelial cells/100 nucleated cells | Many | 1 | 0 |
| Protein g/dL | 4.0 | 4.4 | 3.6 |
| LDH U/L | 1316 | 851 | 687 |
