# Supplementary material for: The CD44high Tumorigenic Subsets in Lung Cancer Biospecimens Are Enriched for Low miR-34a Expression
Source: PLoS One. 2013 Sep 3;8(9):e73195. doi: 10.1371/journal.pone.0073195 (PMC3760902; doi:10.1371/journal.pone.0073195)
Supplement: Supporting Information S2 — Malignant Pleural Effusion (MPE) collection, processing and cell culture. (DOC) [file pone.0073195.s002.doc]

**S-2:** *Malignant Pleural Effusion (MPE) collection, processing and cell culture:*

The process of MPE collection, processing and cell culture is performed routinely in our laboratory and has been described previously (5). In short, MPE specimens were collected from patients at Veterans Affairs-Greater Los Angeles Healthcare System (VAGLAHS) who have undergone a written informed consent process approved by institutional review board. All patients are veterans and MPE specimens are collected by large volume thoracenteses. First, cells and MPE fluid were separated by centrifugation, and then cells were separated by density gradient centrifugation to obtain cells devoid of RBC. The fluid component of MPE is stored at -80^o^C for future use. The cells derived from MPE were then cultured in DMEM-H (HyClone,UT) media supplemented with 20-30% of autologous MPE, plus antibiotics. Several primary cell cultures are established over 3-6 weeks, and aliquots can be frozen in liquid nitrogen for reestablishing cultures for future studies. Adherent primary cultures are detached (Trypsin-EDTA, Sigma, MO) to obtain single cell suspension for live sorting and phenotypic bioassays.
